# Theory of change framework for program evaluation in Health Professional Education

**DOI:** 10.12669/pjms.40.4.8960

**Published:** 2024

**Authors:** Khadija Farrukh

**Affiliations:** 1 Dr. Khadija Farrukh, Head of Department, Department of Medical Education, Bahria University Health Sciences Campus, Karachi, Pakistan. Email: khadijafarrukh2010@hotmail.com

The Theory of Change is a structured, collaborative, participatory and practical approach. It helps to clarify the underlying assumptions, logic behind a program evaluation and how it is expected to bring about change, and how these changes contribute to the desired outcomes. First step in applying theory of change in health professional education is to clearly define the ultimate goals of the medical education program. What changes are you aiming to achieve in the students’ knowledge, skills, problem-solving and attitude. Desired outcomes might include producing competent and compassionate medical professionals who can effectively diagnose and treat patients.^1^ Break down the steps or activities that need to occur to achieve these desired outcomes. Identify the causal linkages and relationships between each step. List the assumptions that underlie your program’s logic, which are the conditions that must be accomplished for the program to progress as expected. Identify the resources and inputs required for the program, such as qualified faculty, appropriate teaching materials, clinical facilities, and administrative support. Determine how you will measure progress and success at each stage of the pathway. What specific key performance indicators will be used at input, output, process, immediate outcome, intermediate outcome and long-term outcome stages to assess whether the program is achieving what is intended to be achieved? Key performance indicators should be specific, measurable, attainable, relevant and actionable. Input indicators like, financial resources, faculty availability, curriculum development and infrastructure. Process indicators can be Teaching and learning methods, Faculty development, assessment and feedback, clinical training, inter-professional education. Output indicators can be students’ scores and knowledge. Immediate outcome are students clinical competence. Intermediate outcome indicator is improved patient outcome. Long-term outcome indicator is improved healthcare in community. Impact indicator is sustainability in community healthcare. A comprehensive evaluation plan that outlines when and how data will be collected to measure the defined indicators.^2^ Consider using both qualitative and quantitative methods, such as surveys, interviews, focus groups. Real-time evaluation and monitoring should be integrated in program evaluation which allows continues gathering of data, and track changes over time. This allows you to make any necessary adjustments based on real-time feedback. Regularly review the collected data to assess whether the assumptions are holding true and whether the program is achieving the desired outcomes. It can be achieved by indicator tracking table or indicator reference sheet. If outcomes are not being met as expected, revisit your Theory of Change and identify potential areas for improvement. Although theory of change seems similar to logic model, but it’s actually quite different as it works at strategic level and incorporate multiple logic models in its mapping. In context of applying theory of change in health professional education a combination of input, process and output indicators can provide comprehensive view of impact of theory of change on health professional education and to determine effective change. This approach will create a structured framework to evaluate your health professional education program’s effectiveness and impact on the students and, ultimately, on patient care.
